# Moral injury and peri- and post-military suicide attempts among post-9/11 veterans

**DOI:** 10.1017/S0033291721005274

**Published:** 2023-05

**Authors:** Shira Maguen, Brandon J. Griffin, Dawne Vogt, Claire A. Hoffmire, John R. Blosnich, Paul A. Bernhard, Fatema Z. Akhtar, Yasmin S. Cypel, Aaron I. Schneiderman

**Affiliations:** 1 San Francisco VA Healthcare System, San Francisco, CA, USA; 2 University of California – San Francisco, San Francisco, CA, USA; 3 Central Arkansas VA Healthcare System, Little Rock, AR, USA; 4 University of Arkansas for Medical Sciences, Little Rock, AR, USA; 5 VA Boston Healthcare System, Boston, MA, USA; 6 Boston University School of Medicine, Boston, MA, USA; 7 VA Eastern Colorado Health Care System, Aurora, Colorado, USA; 8 University of Colorado School of Medicine, Aurora, Colorado, USA; 9 University of Southern California, Los Angeles, CA, USA; 10 VA Pittsburgh Healthcare System, Pittsburgh, PA, USA; 11 Health Outcomes of Military Exposures, Epidemiology Program, Office of Patient Care Services, Veterans Health Administration, Washington, DC, USA

**Keywords:** Suicide, moral injury, posttraumatic stress disorder, depression, veteran, military

## Abstract

**Background:**

Our goal was to examine the association between moral injury, mental health, and suicide attempts during military service and after separation by gender in post-9/11 veterans.

**Methods:**

A nationally representative sample of 14057 veterans completed a cross-sectional survey. To examine associations of exposure to potentially morally injurious events (PMIEs; witnessing, perpetrating, and betrayal) and suicidal self-directed violence, we estimated two series of multivariable logistic regressions stratified by gender, with peri- and post-military suicide attempt as the dependent variables.

**Results:**

PMIE exposure accounted for additional risk of suicide attempt during and after military service after controlling for demographic and military characteristics, current mental health status, and pre-military history of suicidal ideation and attempt. Men who endorsed PMIE exposure by perpetration were 50% more likely to attempt suicide during service and twice as likely to attempt suicide after separating from service. Men who endorsed betrayal were nearly twice as likely to attempt suicide during service; however, this association attenuated to non-significance after separation in the fully adjusted models. In contrast, women who endorsed betrayal were over 50% more likely to attempt suicide during service and after separation; PMIE exposure by perpetration did not significantly predict suicide attempts before or after service among women in the fully adjusted models.

**Conclusions:**

Our findings indicate that suicide assessment and prevention programs should consider the impact of moral injury and attend to gender differences in this risk factor in order to provide the most comprehensive care.

Preventing veteran suicide is a national priority due to disproportionate rates of veteran suicides compared to the general population. The suicide rate among veterans is 1.5 times that of non-veteran adults, even after adjusting for age and sex (U.S. Department of Veterans Affairs, 2019). More than 6000 veteran suicides have occurred each year over the last decade (U.S. Department of Veterans Affairs, 2019), and the majority of these suicide deaths occur among those not using Veterans Health Administration (VHA) services in the year before death (U.S. Department of Veterans Affairs, 2020). Multiple initiatives have been implemented to enhance understanding of factors associated with the greatest risk for suicide and algorithms have been developed to identify high-risk veterans using VHA services (Kessler et al., 2017; McCarthy et al., 2015). These initiatives demonstrate that a combination of demographic and clinical factors place veterans at increased risk. While risk factors such as prior suicide attempts and mental health diagnoses such as depression, substance use disorder, and PTSD are well-documented (Conner et al., 2014; LeardMann et al., 2013; Nock et al., 2013; Pruitt et al., 2019) and are easily identifiable from individuals' medical records, there are other factors that may not be as straightforward to identify and may be associated with barriers to seeking mental health care (e.g. Nazarov, Fikretoglu, Liu, Richardson, & Thompson, 2020). For example, one study found that veterans at risk for moral injury were less likely than those not at risk to seek mental health care (Nazarov et al., 2020).

Moral injury is an emerging construct that has received growing attention over the last decade and is defined as ‘perpetrating, failing to prevent or bearing witness to acts that transgress deeply held moral beliefs or values’ (Litz et al., 2009). Moral injury can manifest if a veteran feels like they have crossed a line of their personal or shared values while serving in the war, and consequently experiences profound guilt, shame, withdrawal, a failure to self-forgive, self-sabotaging behaviors, and functional impairment (Griffin et al., 2019; Litz et al., 2009). Additionally, a moral injury may occur in the context of being betrayed, specifically when there has been a betrayal of accepted norms of morality by a person in legitimate authority in a high-stakes situation (Shay, 2014). Potentially morally injurious events (PMIEs) may include acts of commission (e.g. killing in war) and omission (e.g. failing to save someone), witnessing serious injury or death and dying, or betrayal.

Growing evidence suggests that moral injury in veterans may be a distinct risk factor for suicide, separate from PTSD and other mental health conditions. Several studies have found that both exposures to PMIEs and moral injury are associated with suicidal ideation and attempts (Ames et al., 2019; Kelley et al., 2019; Kline, Weiner, Interian, Shcherbakov, & St. Hill, 2016; Levi-Belz, Dichter, & Zerach, 2022; Maguen et al., 2012; Wisco et al., 2017). In one study, suicidal ideation was nearly double for those who endorsed greater killing experiences in war, even after adjusting for the effects of PTSD, depression, substance use, and general combat (Maguen et al., 2012). Notably, although PTSD and moral injury are distinct constructs, the combination further increases the risk for suicidal behavior (Bryan, Bryan, Roberge, Leifker, & Rozek, 2018).

Gender is one important factor that has been differentially linked to both veterans' suicidal behavior and their experiences of moral injury. Both quantitative and qualitative studies have found gender differences in suicide behaviors and suicide-related outcomes among veterans (Denneson et al., 2020; Gradus, King, Galatzer-Levy, & Street, 2017; Hoffmire, Kemp, & Bossarte, 2015; Horwitz, Smith, Held, & Zalta, 2019; Ursano et al., 2018), with one recent study revealing precursors to women's suicide attempts that were consistent with hallmark symptoms of moral injury (e.g. shameful, tainted, and worthless), in contrast with contributors for men, which were more likely to reflect frustration with others and feeling like they had reached a personal limit (Denneson et al., 2020). In addition, a recent study found important gender differences in the manifestation and expression of moral injury, with some evidence that the relationship between the type of moral injury exposure and functional outcomes varies by gender (Maguen et al., 2020). Maguen et al. (2020) found that perpetration (acts of omission and commission) was most consistently associated with functional impairment across domains for men, whereas betrayal was most consistently associated with functional impairment for women. Taken together, this research highlights the importance of examining associations between moral injury and suicide behaviors separately for men and women.

Additional factors that are important to consider when studying the relationship between moral injury and suicidal behavior are the timing of suicide behavior (peri- or post-military) as well as an examination of risk factors across the life course. Risk for peri- and post-military suicide behavior has been shown to differ in important ways (Hoffmire et al., 2021a), yet there is a paucity of research on factors that confer differential risk based on timing of military service, necessitating further examination. Furthermore, the transition period between peri- and post-military service is a time of heightened risk for suicide (Ravindran, Morley, Stephens, Stanley, & Reger, 2020). Further research is also warranted to better understand risk and resilience factors during this time. Finally, examining risk factors across the life course (i.e. pre-, peri- and post-military) including pre-military risk factors like early childhood trauma and pre-military suicidal ideation and attempts; military factors like military sexual trauma (MST), combat exposure, and exposure to PMIEs; and post-military factors like mental health symptoms ensures a broad perspective (Nock, Ramirez, & Rankin, 2019).

To address these knowledge gaps, in the current study we investigated post-9/11 veterans' experiences of PMIEs related to acts of perpetration, witnessing, and betrayal and examined relationships with peri- and post-military suicide attempts by gender. We hypothesized that PMIE exposure related to both perpetration and betrayal would be associated with peri- and post-military suicide attempts, and that similar to prior studies examining gender differences in moral injury, the relationship between PMIE exposure by perpetration and suicidal attempts would be strongest for men.

## Methods

### Participants and procedure

The Comparative Health Assessment Interview (CHAI) Research Study is a cross-sectional survey that focused on the health and well-being of post-9/11 veterans and non-veterans. The current analysis focuses on the veteran subsample. CHAI utilized a stratified probability-based sampling frame of post-9/11 veterans from the U.S. Veterans Eligibility Trends and Statistics (USVETS) dataset, which includes information on all separated veterans who had been active duty or had a period of active service (as a member of the Reserves or National Guard) between September 2001 and June 2015.

The sampling design was used to select veterans based on whether they were deployed for service during military operations following 9/11. Deployed veterans were sampled using stratification by service branch (Army, Air Force, Marines, Navy) and component (Active Duty, Reserves/National Guard), and whether their first activation was before or after 9/11, with females oversampled (30% of sample). Non-deployed veterans were then matched across all strata. Veterans were invited to participate by mail and then given the option to complete the survey online or by phone. Veterans who did not complete the online survey received follow-up calls.

Data were collected between April 18, 2018 and August 10, 2018. Veterans were recruited by mail (invitation, two reminders) to complete the survey via either a secured online survey or a computer-assisted telephone interview, with additional reminder calls to non-responders. Invitations included a $1 pre-incentive; respondents received a $50 post-incentive. The response rate for veterans was 40%, similar to response rates of other recent population-based surveys of veterans (Bastian et al., 2014; Eber et al., 2013; Street, Gradus, Giasson, Vogt, & Resick, 2013).

Veterans' (*N* = 15 166) gender, age, race, sexual orientation, marital status, parental status, the highest level of education attained, branch of military service, and rank at discharge are reported in Table 1. Gender (stratification variable) was defined by self-report (man, woman, transgender man [female to male (FTM)], transgender woman [male to female (MTF)], gender non-conforming, and different identity. Transgender respondents (*n* = 18) were categorized according to their gender identity [FTM = man (*n* = 11), MTF = woman (*n* = 7)]. Where self-reported gender was missing (*n* = 39), we replaced the missing value with the sex assigned at birth from the original sampling frame [male = man (*n* = 32), female = woman (*n* = 7)]. Those who reported gender as non-conforming or different identity (*n* = 28) were excluded from the analysis. An additional inclusion criterion for the current study was that respondents either (1) reported no exposure to a traumatic event (*n* = 1188) or (2) identified a most currently distressing traumatic event (*n* = 12 869). Thus, the total number of cases analyzed for this study included 14 057 veterans.

**Table 1.** Weighted percentages for demographic and military-related characteristics

	Full sample	Men (82.3%)	Women (17.7%)
Age			
18–29 years	18.6	17.9	22.0
30–39 years	44.2	43.6	47.0
40–49 years	19.1	19.3	18.2
50+ years	18.1	19.2	12.8
Race/Ethnicity			
White Non-Hispanic	66.2	68.7	55.0
Black Non-Hispanic	12.8	11.0	21.2
Other Non-Hispanic	3.8	3.8	3.5
Multiracial Non-Hispanic	5.8	5.7	6.3
Hispanic	11.4	10.8	14.0
Marital status			
Never married/Divorced/Widowed	36.6	34.6	45.7
Married/Civil union	63.5	65.4	54.3
Parental status			
Parenting responsibility	49.5	47.7	57.8
No parenting responsibility	50.5	52.3	42.2
Sexual orientation			
Heterosexual	95.6	97.2	87.9
Sexual minority	4.5	2.8	12.1
Highest education attained			
Some college or lower	58.3	59.7	51.9
Assoc. degree or higher	41.7	40.3	48.2
Branch of service			
Army	50.5	50.0	52.6
Navy/Coast Guard	17.9	17.7	18.9
Marines	13.3	15.0	5.6
Air Force	18.3	17.3	23.0
Rank			
Enlisted	88.1	87.9	88.6
Officer/Warrant officer	11.9	12.1	11.4
Warzone deployed			
Yes	56.9	60.6	39.7
No	43.1	39.4	60.3
Military sexual trauma			
Yes	11.9	5.1	43.2
No	88.1	94.9	56.8
Adverse childhood experiences			
0	23.5	23.8	21.8
1–2	33.0	33.7	29.6
3–4	18.7	18.6	19.0
5–6	11.2	10.8	13.1
>6	13.6	13.0	16.5

*Note.* Values given as percentages. Participants were men (*n* = 8809) and women (*n* = 5248) veterans weighted to the population of post-9/11 veterans activated since 10/1/2001 and separated by 6/30/2015 (*N* *=* *3 691 536*). In the weighted sample, 82.3% of participants identified as a man and 17.7% identified as a woman.

Weighted percentages by demographic and military characteristics are reported in Table 1. Veterans identified as men (82.3%) and women (17.7%). The largest proportions of the sample were between 30 and 39 years old (44.2%), of non-Hispanic white race/ethnicity (66.2%), heterosexual (95.6%), currently married or in a civil union (63.5%) and reported less than an associate degree (58.3%). Veterans were also most likely to report service in the Army (50.5%) relative to other branches of the Armed Forces and to have been of enlisted *v.* officer rank (88.1%). Over half of respondents reported being deployed to a warzone (56.9%). Warzone deployed men reported either zero (15.4%; i.e. combat deployed but not to Iraq/Afghanistan), one (44.6%), two (22.6%), or three or more (17.4%) deployments to either Iraq or Afghanistan; warzone deployed women reported either zero (23.9%), one (50.3%), two (17.0%), or three or more (8.7%) deployments to either Iraq or Afghanistan. Roughly 5% of men and 40% of women endorsed MST. Over 75% of veterans endorsed exposure to at least one adverse childhood event.

### Measures

Our primary outcome was suicidal self-directed violence. We administered a modified version of the Columbia-Suicide Severity Rating Scale (C-SSRS; Katz, Barry, Cooper, Kasprow, & Hoff, 2020; Matarazzo et al. 2019; Posner et al. 2011) to assess the prevalence of active suicidal ideation (‘Have you ever actually had any thoughts of killing yourself?’) and suicide attempt (‘Have you ever made a suicide attempt’). Respondents who reported having thought about or attempted to kill themselves in their lifetime were further asked whether the experience occurred pre-, peri-, or post-military service and were given the option to select all that applied.

The Moral Injury Events Scale (MIES; Nash et al., 2013) was used to assess exposure to and subjective distress from PMIEs encountered in the military setting. This scale includes nine items that assess PMIE exposure by (1) witnessing, (2) perpetrating (through acts of commission or omission), or (3) being betrayed. Sample items include: ‘I acted in ways that violated my own moral code or values.’ And ‘I am troubled by having acted in ways that violated my own morals or values.’ Participants rated each item using a 6-point response format (1 = *strongly disagree* to 6 = *strongly agree*). Internal consistency was excellent (*α* = 0.908). To enhance the interpretability of these findings, we created binary variables for witnessing, perpetrating, and being betrayed by collapsing responses into two categories: those who reported slight, moderate, and strong *disagreement* with items and those who reported slight moderate, and strong *agreement* with items (Maguen et al., 2020). Furthermore, we used the three-factor structure proposed by Bryan et al. (2016) to assess exposure to a PMIE by witnessing, perpetrating, and being betrayed. Thus, positive response on the witnessing variable indicates an ‘agree’ response to either or both items 1 and 2, positive response on the perpetrating variable indicates an ‘agree’ response to one or more of items 3 through 6, and a positive response on the being betrayed variable indicates an ‘agree’ response to one or more of items 7 through 9.

Respondents also completed measures of psychiatric and behavioral problems. The Posttraumatic Stress Disorder (PTSD) Checklist (PCL-5; Weathers et al., 2013) is a self-report questionnaire comprised of 20 items that correspond to the Diagnostic and Statistical Manual-5 (DSM-5) symptom criteria for PTSD. Respondents indicated how much they were bothered (0 = not at all, 4 = extremely) by each symptom in the prior month. Item responses were summed to create a total score, and the recommended cutoff of 33 was used to indicate a positive screen for PTSD (Blevins, Weathers, Davis, Witte, & Domino, 2015). Only respondents who endorsed experiencing a potentially traumatic event in their lifetime (PTE; e.g. combat, physical/sexual assault, life-threatening injury/illness) and were able to identify their most PTE were administered the PCL-5 items. We assigned a zero-sum score for those who reported no exposure to a Criterion A stressor (*n* = 1193). Internal consistency was excellent (*α* = 0.97).

The Patient Health Questionnaire – 9 (PHQ-9; Kroenke, Spitzer, and Williams, 2001) is a nine-item self-administered questionnaire based on the DSM-5 criteria for Major Depressive Disorder. Respondents indicated the frequency with which they were bothered by each symptom during the previous 2 weeks using a 4-point response format (0 = *not at all*, 3 = *nearly every day*). Item scores were summed to create a total score, and the recommended cutoff of 10 was used to indicate a positive screen for depressive disorder. Internal consistency was excellent (*α* = 0.92).

The Generalized Anxiety Disorder 7-item scale (GAD-7; Spitzer, Kroenke, Williams, & Löwe, 2006) is a self-administered instrument based on the DSM-IV criteria for Generalized Anxiety Disorder. Respondents indicated the frequency with which they were bothered by each symptom during the previous 2 weeks using a 4-point response format (0 = *not at all*, 3 = *nearly every day*). Item scores were summed to create a total score, and the recommended cutoff of 10 was used to indicate a positive screen for anxiety disorder. Internal consistency was excellent (*α* = 0.94).

The Alcohol Use Disorder Identification Test – Concise (AUDIT-C; Bush, Kivlahan, McDonell, Fihn,& Bradley, 1998) is a screening tool that identifies persons likely to have active alcohol dependence or abuse. Respondents indicated how often they consumed alcohol (0 = *never*, 4 = *four or more times per week*), how many drinks they consumed in a typical day (0 = *none/1–2 drinks*, 4 = *ten or more drinks*), and how often they had six or more drinks on one occasion (0 = *never*, 4 = *daily or almost daily*) during the past year. Item scores were summed to create a total score, and the recommended cutoff of 3 for women and 4 for men was used to identify probable alcohol use disorder. Due to branching logic in the survey, only those who reported some use of alcohol in their lifetime were administered the AUDIT-C items. We imputed a zero-sum score for those who reported never using alcohol (*n* = 1129). Internal consistency was acceptable (*α* = 0.74).

To assess the history of exposure to potentially traumatic events that are common among veterans, respondents also responded to questions about warzone deployments, MST, and adverse childhood experiences (ACEs). Warzone deployment was assessed using a single item on which Veterans identified if they (1) had not deployed, (2) deployed but not to a warzone, (3) deployed to war in Iraq/Afghanistan, and (4) deployed to war but not in Iraq/Afghanistan. To be inclusive of those deployed to war in any combat era, we collapsed the categories to create a binary variable representing any warzone deployment *v.* no warzone deployment. We also assessed a number of deployments specifically to Iraq or Afghanistan.

We assessed MST using the VHA electronic medical record MST screener. The MST screener contains two items: ‘When you were in the military: (a) Did you ever receive unwanted, threatening, or repeated sexual attention (for example, touching, cornering, pressure for sexual favors, or inappropriate verbal remarks, etc.)?; (b) Did you have sexual contact against your will or when you were unable to say no (for example, after being forced or threatened or to avoid other consequences)?’ These items have been validated against clinical interviews and psychometrically sound questionnaires (Kimerling, Gima, Smith, Street, & Frayne, 2007). Respondents answered each question using a 3-point option (1 = *Yes,* 2 = *No*, 3 = *Decline*). We transformed the original variable to create a binary variable representing an affirmative ‘yes’ response to either or both questions *v.* a ‘no’ response to both questions. Decline responses were coded as missing.

The history of ACEs in the CHAI sample is reported elsewhere, including the association between ACES and self-directed suicidal violence (Blosnich et al., 2021). In accordance with prior research, we replicated the approach described by Blosnich et al., to transform 23 variables that assessed exposure to an array of potentially traumatic experiences (e.g. natural disaster, physical assault, food/housing insecurity, etc.) before the age of 18 into a 5-category ordinal variable representing the cumulative frequency of ACEs (0, 1–2, 3–4, 5–6, >6 ACEs). We included one additional item in the ACEs composite that was inadvertently omitted from the Blosnich study (i.e. ‘learned about violent or accidental death/serious injury to close relative or friend’).

### Data analysis

Inspection across all study variables revealed that 8% of cases were missing a value on at least one of twenty-two possible variables. Less than 1% of the total possible values were missing. To utilize all available data without biasing parameter estimates, we performed multiple imputations with fifteen imputed datasets from which we derived coefficient estimates and standard errors that we pooled to obtain final results (Pedersen et al., 2017). All analyses were weighted to account for the complex sampling design and used Taylor series approximation (linearization) for variance estimation. Weights included a base sampling weight, a nonresponse-bias adjustment, and a calibration to the population frame on gender, branch, component, geographic stratum, deployment, and pre-/post-9/11 activation (though all served on active duty or had periods of activation post-9/11, a subset had also been on active duty or activated prior to 9/11).

Preliminary analyses included bivariate comparisons between men and women veterans in terms of the prevalence of PMIE exposure and mental health problems using weighted simple logistic regression. To further assess whether veterans exposed to PMIEs by witnessing, perpetrating, and being betrayed were more likely to attempt suicide during and after military service, we conducted a series of weighted multiple logistic regressions. Partially adjusted models accounted for variance in suicide attempts explained by exposure to a PMIE, controlling for covariation between PMIE exposure by witnessing, perpetrating, and being betrayed but no other covariates. Fully adjusted models also accounted for variance in suicide attempts explained by demographic and military-related characteristics (i.e. age, race and ethnicity, sexual orientation, marital status, parental status, a branch of service, rank, and the highest level of education, history of warzone deployment, MST, and adverse childhood events), current mental health status (i.e. traumatic stress, depression, anxiety, hazardous alcohol use), and pre-military history of suicidal ideation and suicidal self-directed violence.

Adjusted odds ratios (AOR) with 95% confidence intervals (95% CI) are reported for the fully adjusted models in online Supplementary Table S1. We used the Zhang and Yu (1998) method to approximate the adjusted relative risk (ARR) based on the AOR and percentage of the sample who attempted suicide in the unexposed subgroup (e.g. those who screened negative for PTSD; online Supplementary Table S2). Finally, we report estimates of the association between PMIE exposure and suicide attempt in online Supplementary Table S3 without controlling for covariates (unadjusted), controlling only for demographic and military-related characteristics (partially adjusted), and controlling for current mental health status and history of suicidal thoughts and behaviors (fully adjusted). Analyses were conducted using SAS Enterprise Guide version 7.1.

## Results

As is shown in Table 2, we observed gender differences in the weighted prevalence of suicide attempts, such that women reported a higher prevalence of suicide attempts than men prior to (women = 6.1%; men = 1.4%), during (women = 5.7%; men = 2.6%), and after (women = 5.9%; men = 3.5%; Table 2) military service. Overall, 12.4% of women and 5.5% of men reported at least one suicide attempt. Of the veteran men and women who reported at least one attempt, 47.7% of men and 39.8% of women reported one attempt, 24.1% of men and 23.5% of women reported two attempts, and 28.2% of men and 36.7% of women reported three or more attempts.

**Table 2.** Weighted bivariate comparisons of study variables by gender

	Full sample	Men	Women	OR	95% CI
Witnessing				**1.59**	**1.46–1.72**
Agree	43.1	41.0	52.5		
Disagree	56.9	59.0	47.5		
Perpetrating				1.18	1.07–1.30
Agree	21.8	21.3	24.1		
Disagree	78.2	78.7	75.9		
Being betrayed				**1.61**	**1.49–1.75**
Agree	40.2	38.1	49.8		
Disagree	59.8	61.9	50.2		
Posttraumatic stress disorder				1.45	1.32–1.59
Positive	20.9	19.8	26.3		
Negative	79.1	80.2	73.7		
Depressive disorder				1.37	1.25–1.50
Positive	25.4	24.3	30.5		
Negative	74.6	75.8	69.5		
Anxiety disorder				1.47	1.34–1.61
Positive	22.1	20.8	27.9		
Negative	77.9	79.2	72.1		
Alcohol use disorder				0.83	0.77–0.90
Positive	38.0	38.7	34.5		
Negative	62.0	61.3	65.5		
Pre-military suicide ideation				**2.19**	**2.18–2.21**
Yes	7.9	6.7	13.5		
No	92.1	93.3	86.5		
Pre-military suicide attempt				**4.64**	**4.57–4.70**
Yes	2.2	1.4	6.1		
No	97.8	98.6	93.9		
Peri-military suicide attempt				**2.29**	**2.26–2.32**
Yes	3.1	2.6	5.7		
No	96.9	97.4	94.3		
Post-military suicide attempt				**1.77**	**1.75–1.79**
Yes	3.9	3.5	5.9		
No	96.1	96.6	94.1		

*Note.* Values given as percentages. Abbreviations include odds ratio (OR) and 95% confidence interval (95% CI). Participants were men (*n* = 8809) and women (*n* = 5248) veterans weighted to the population of post-9/11 veterans activated since 10/1/2001 and separated by 6/30/2015 (*N* *=* *3 691 536*). The reference category is male (*v.* female). All mental health disorders are assessed by screening measures. PMIE exposure was assessed using the Moral Injury Events Scale (MIES, Nash et al., 2013) and modeled by witnessing (0 = disagree, 1 = agree), perpetrating (0 = disagree, 1 = agree), and being betrayed (0 = disagree, 1 = agree). Bolding indicates ORs of at least small effect size (OR ≥ 1.52; Chen, Cohen, & Chen, 2010).

Women were more likely to endorse PMIE exposure by witnessing (women = 52.5%; men = 41.0%) and being betrayed (women = 49.8%; men = 38.1%); there was no evidence of a gender difference in PMIE exposure by perpetrating (women = 24.1%; men = 21.3%). A sizable portion of veterans screened positive for posttraumatic stress (20.9%), depressive (25.4%), anxiety (22.1%), and alcohol use (38.0%) disorders; gender differences observed for mental health problems were statistically significant but marginal in effect size. Women (13.5%) more than men (6.7%) endorsed a pre-military history of suicidal ideation.

### Veteran men

The partially adjusted models controlling only for covariance between PMIE exposure by witnessing, perpetrating, and being betrayed revealed that men who endorsed PMIE exposure by perpetrating were more than twice as likely to attempt suicide during (ARR = 2.00, 95% CI 1.42–2.82) and after (ARR = 2.63, 95% CI 1.91–3.62) military service. The partially adjusted models also showed that men who endorsed being betrayed were two to three times more likely to attempt suicide during (ARR = 3.21, 95% CI 2.14–4.79) and after (ARR = 2.35, 95% CI 1.67–3.31) military service. No evidence suggested that witnessing others' immoral acts contributed to the likelihood of suicide attempts during (ARR = 0.90, 95% CI 0.62–1.31) or after (ARR = 0.97, 95% CI 0.67–1.39) military service among men.

As is shown in the fully adjusted model presented in Table 3, when also controlling for current mental health status, history of suicidal ideation and attempt, and differences in demographic and military-related characteristics, men who reported perpetration were still 50% more likely to attempt suicide during service (ARR = 1.52, 95% CI 1.05–2.18) and twice as likely to attempt suicide after separation (ARR = 2.01, 95% CI 1.43–2.80) relative to those who denied perpetration. The fully adjusted models indicated further that men who reported being betrayed were nearly twice as likely to attempt suicide during service relative to those who reported no betrayal (ARR = 1.90, 95% CI 1.25–2.87); however, the association between betrayal and suicide attempt attenuated after separation from service (ARR = 1.29, 95% CI 0.89–1.86) when accounting for other predictors of suicide attempt in the fully adjusted models.

**Table 3.** Fully adjusted relative risk and confidence limits weighted gender-stratified models predicting suicide attempt

	Men				Women			
	Peri-military attempt		Post-military attempt		Peri-military attempt		Post-military attempt	
	ARR	95% CI	ARR	95% CI	ARR	95% CI	ARR	95% CI
Witnessing	0.71	0.47–1.06	0.76	0.54–1.11	1.39	0.90–2.13	0.91	0.60–1..38
Perpetrating	**1.52**	**1.05–2.18**	**2.01**	**1.43–2.80**	1.24	0.89–1.72	1.26	0.87–1.80
Being betrayed	**1.90**	**1.25–2.87**	1.29	0.89–1.86	**1.56**	**1.00–2.41**	**1.62**	**1.06–2.44**
PTSD	**1.71**	**1.10–2.65**	1.45	0.98–2.13	**2.02**	**1.40–2.91**	**2.06**	**1.38–3.04**
Depressive disorder	**2.20**	**1.31–3.67**	**3.44**	**2.21–5.28**	**2.00**	**1.27–3.09**	**1.75**	**1.12–2.74**
Anxiety disorder	1.39	0.87–2.19	1.31	0.86–2.00	1.07	0.64–1.61	1.07	0.70–1.61
Alcohol use disorder	1.03	0.47–1.43	1.15	0.86–1.53	1.21	0.90–1.60	1.22	0.89–1.65
Attempt pre-military	**3.12**	**1.58–5.95**	**4.46**	**2.48–7.65**	**2.62**	**1.59–4.19**	**2.60**	**1.65–4.00**
Ideation pre-military	**2.39**	**1.49–3.77**	**1.68**	**1.07–2.60**	**2.25**	**1.42–3.52**	**3.21**	**2.14–4.71**

*Note.* Participants were men (*n* = 8809) and women (*n* = 5248) veterans weighted to the population of post-9/11 veterans activated since 10/1/2001 and separated by 6/30/2015 (*N* *=* *3 691 536*). Abbreviations include Moral Injury Events Scale (MIES; Nash et al., 2013) and posttraumatic stress disorder (PTSD). Independent variables were binary (0, 1). Demographic and military-related characteristics were included in the model but excluded from the table to enhance readability (see also, online Supplementary Table S1). Class variables were effect coded prior to analysis including age (ref: 20s), branch of service (ref: Army), and number of adverse childhood events (ref: 0). Bolding indicates at least small effect size.

Overall, current depressive symptoms, pre-military history of ideation and attempt, and PMIE exposure by perpetration were the most consistent predictors of increased risk for suicide attempts among men during and after military service. Current posttraumatic stress symptoms and being betrayed also predicted a greater likelihood of suicide attempts during service. Critically, PMIE exposure by perpetration contributed to suicide attempts during and after military service beyond the variance explained by other variables entered into the fully adjusted models. Betrayal explained additional variance in suicide attempts during but not after service in the fully adjusted models among men.

### Veteran women

Next, the partially adjusted models revealed that women who endorsed being betrayed were more than twice as likely to attempt suicide during service (ARR = 2.54, 95% CI 1.73–3.72) and after separation (ARR = 2.33, 95% CI 1.62–3.31) relative to those who denied betrayal. Women who endorsed PMIE exposure by perpetrating were also roughly 80% more likely to attempt suicide during (ARR = 1.78, 95% CI 1.31–2.40) and after (ARR = 1.78, 95% CI 1.29–2.45) military service. In contrast to men, women who witnessed others' immoral acts were also about 60% more likely to attempt suicide during (ARR = 1.66, 95% CI 1.14–2.41) but not after military service (ARR = 1.08, 95% CI 0.73–1.56).

As is shown in Table 3, women who endorsed being betrayed remained more than 50% more likely to attempt suicide during service (ARR = 1.56, 95% CI 1.00–2.41) and after separation (ARR = 1.62, 95% CI 1.06–2.44) relative to those who denied betrayal in the fully adjusted models. However, PMIE exposure by perpetrating and witnessing were not significant predictors of suicide attempts during or after military service among women, when controlling for current mental health status, history of suicidal ideation and attempt, and differences in demographic and military-related characteristics.

Overall, the most consistent correlates of suicide attempts during and after service in women were posttraumatic stress symptoms, depressive symptoms, pre-military history of ideation and attempt, and being betrayed. Women who screened positive for MST were also more likely to attempt suicide during service. Whereas betrayal explained incrementally more variance in suicide attempts during and after military service among women after accounting for other predictors of suicide risk, PMIE exposure by witnessing or perpetration did not explain additional variance in risk of a suicide attempt among women in the fully adjusted models.

## Discussion

This is the first study to examine the relationship between PMIE exposure and suicide attempts separately for male and female post-9/11 veterans. Importantly, associations varied for women and men and based on whether the suicide behavior occurred during or after military service. This improved understanding of the impact of gender and timing of suicide behavior can assist with assessing risk and tailoring treatment for veterans across the life course.

When considering all risk factors in a fully adjusted model, PMIE exposure, and perpetration in particular, was a significant predictor of men's *suicide attempts peri- and post-military service*. This finding corroborates several studies that have demonstrated that suicide ideation and attempts are higher in individuals who reported killing or feeling responsible for the death of another during warfare (LeardMann et al., 2021; Maguen et al., 2012). This is important because exposure to PMIEs and their psychological aftermath are typically not assessed in military suicide risk assessments (Zuromski et al., 2019). This finding supports the value of clinical education and training and intervention delivery concerning morally injurious incidents as a prevention strategy, particularly among men who may be at heightened risk of suicide behaviors following these incidents. Related, improving assessment for PMIE exposure and moral injury is also critical, given that veterans will often keep these exposures private unless asked directly in the context of other exposures (Maguen & Burkman, 2013). Asking about a wide range of exposures helps create an accepting environment and conveys the message that discussing difficult topics like killing in war is acceptable within the therapeutic context.

Multiple interventions for moral injury may provide some relief, above and beyond evidence-based treatments for depression and PTSD. For example, Impact of Killing (IOK) is a treatment that targets perpetration-based moral injury by directly addressing the mental health IOK as well as self-forgiveness, self-compassion and the psychosocial and spiritual impact of moral injury (Maguen et al., 2017; Purcell, Griffin, Burkman, & Maguen, 2018). Other treatments that are focused on moral injury include Adaptive Disclosure, Trauma-Informed Guilt Reduction Therapy, Building Spiritual Strength, and Acceptance and Commitment Therapy for Moral Injury (Gray et al., 2012; Harris et al., 2011; Nieuwsma et al., 2015; Norman, Wilkins, Myers, & Allard, 2014). Each of these has either been tested in pilot randomized controlled trials (RCTs) or open trials, and all have larger RCTs underway. Importantly, given that there are residual symptoms following PTSD evidence-based psychotherapies, including guilt due to actions that had to be taken during the traumatic event (Larsen, Fleming, & Resick, 2019), interventions targeting moral injury may help close this residual symptom gap.

We also found that betrayal was associated with suicide behavior peri-military for men and peri- and post-military for women, highlighting the importance of assessing for betrayal. Individuals in the military may feel betrayed by leadership or by fellow soldiers and these experiences have far-reaching impacts. For example, perceptions of institutional betrayal are associated with increased odds of attempting suicide (Monteith, Bahraini, Matarazzo, Soberay, & Smith, 2016b); additionally, betrayal can be linked to barriers to treatment (Holliday & Monteith, 2019). Furthermore, if individuals in the military feel betrayed by leadership's actions, it can cause demoralization, hopelessness and thwarted belongingness, thus contributing to suicide behaviors (e.g. Van Orden et al., 2010).

Similarly, if veterans experience betrayal from fellow service members whom they entrust with their lives, this can also lead to a spiral of adverse mental health symptoms, particularly in the context of MST. Indeed, in this study, MST was associated with suicide behavior peri-military, further corroborating these relationships. Evidence links MST and suicide risk (Bryan, Bryan, & Clemans, 2015; Forkus et al., 2021; Monteith et al., 2016a, 2016b); however, closely delineating that the association may be strongest peri-military service is an important addition to the literature and can help better target timing of assessment and interventions as needed. Importantly, one prior study did not find that betrayal was associated with suicide attempts, but this sample was predominantly composed of older male veterans and the outcome was lifetime suicide attempts rather than suicidal behavior experienced during and after military service (Nichter, Norman, Maguen, & Pietrzak, 2021).

Not surprisingly, pre-military ideation and attempts were strong and consistent predictors of suicidal behavior peri- and post-military service, as were depression symptoms, with depression demonstrating a strong effect size and consistent relationship with suicide attempts across gender and the life course (Nock et al., 2014; Levey et al., 2019). Associations between prior suicide thoughts and behavior, mental health symptoms and suicide attempts have consistently been shown in the broader epidemiologic literature on attempted suicide (Nock et al., 2008; Ribeiro et al., 2016). We also found that PTSD was associated with suicide attempts for women, both peri- and post-military service, but for men only during military service. Prior studies have found a strong association between prior suicide attempts and PTSD (e.g. LeardMann et al., 2021). However, it is important to note that in a systematic review examining PTSD and suicide behaviors, while most studies found an association between PTSD and suicide attempt, the authors noted that overall, studies did not adequately sample women or report rates or associations separately based on gender (Holliday et al., 2020). Our finding may shed light on the importance of examining these relationships by gender rather than adjusting for gender.

When examining PMIE exposure, we found that women reported higher rates of PMIE exposure due to witnessing and betrayal, but that there were no gender differences with respect to perpetration (either acts of omission or commission). Importantly, women also reported higher suicide attempt rates before, during, and after military service than men. Additionally, women who attempted suicide tended to have a greater number of attempts, compared to men. These gender findings are consistent with prior epidemiological studies in the general population (Nock et al., 2008) as well as studies that have found that women veterans are more likely to report suicide attempts (Ursano et al., 2015; Hoffmire et al., 2021a). Although reasons for the higher rates in women remain unclear and more research is underway to better understand these gender findings (Hoffmire et al., 2021b), one recent qualitative study found that when women veterans describe reasons for their suicide attempts they discuss themes of self-concept, social power, and relationships (Denneson et al., 2020); more specifically, they highlight negative self-evaluation and internalizing behaviors, while for men the blame is often more external, related to finding fault in the world around them, which may account for some of these differences.

Several limitations of our study should be noted. Our sample included post-9/11 veterans and may not generalize to veterans of other eras. There is some evidence that findings on the impact of moral injury on suicidal outcomes may vary according to era or time since event, with a stronger relationship between moral injury and suicide for younger veterans who experienced PMIEs more recently (Nichter et al., 2021). Next, we were able to adjust for current symptoms given that they were measured during the same time period as moral injury and pre-military history of suicide ideation and attempt; however, we did not adjust for lifetime mental health diagnoses given that these were measured dichotomously over the lifetime and it was not clear whether these preceded or followed our suicide attempt dependent variables. Additional limitations include the survey response rate, and the cross-sectional nature of the study. Finally, measures of moral injury may conflate exposure and the downstream effects of those exposures to some extent; better measures are needed. Despite this, we used the most robust measure of moral injury available.

Overall, findings of this study suggest that even after accounting for a host of factors including mental health symptoms, PMIE exposure due to perpetration is a risk factor for men's suicide attempts during and after military service, and PMIE exposure due to betrayal is a risk factor for both women's and men's suicidal attempts during military service, but only women's suicidal attempts after service. Other important suicide risk factors include pre-military suicidal ideation and attempts, depression, PTSD symptoms, and MST. Of note, for men, while the relationship between PTSD and post-military suicide attempts weakened and was no longer significant (from peri- to post-military), the relationship between PMIE exposure by perpetration and post-military suicide attempt became stronger. Any comprehensive suicide assessment and prevention program should account for the impact of moral injury to ensure that veterans are thoroughly assessed for critical risk factors.

## Supplementary material

For supplementary material accompanying this paper visit https://doi.org/10.1017/S0033291721005274. click here to view supplementary material
