# Supplementary material for: Moral injury and peri- and post-military suicide attempts among post-9/11 veterans
Source: Psychol Med. 2022 Jan 17;53(7):3200–9. doi: 10.1017/S0033291721005274 (PMC10235653; doi:10.1017/S0033291721005274)
Supplement: Supplementary file 1 [file S0033291721005274sup001.docx]

| Supplemental Table 1. *Odds Ratios and Confidence Limits for Fully Adjusted Weighted Models predicting Suicide Attempt by Gender* | | | | | | | | | | | |
| --- | --- | --- | --- | --- | --- | --- | --- | --- | --- | --- | --- |
|  | Men | | | | |  | Women | | | | |
|  | Peri-Military Attempt | |  | Post-Military Attempt | |  | Peri-Military Attempt | |  | Post-Military Attempt | |
|  | OR | 95% CI |  | OR | 95% CI |  | OR | 95% CI |  | OR | 95% CI |
| Age 30s v 20s | 0.95 | 0.74-1.23 |  | 1.44 | 1.15-1.80 |  | 0.88 | 0.70-1.11 |  | **1.63** | **1.26-2.11** |
| Age 40s v 20s | 0.92 | 0.67-1.26 |  | 1.06 | 0.79-1.42 |  | 1.18 | 0.89-1.57 |  | 1.29 | 0.92-1.81 |
| Age 50s+ v 20s | 0.99 | 0.68-1.43 |  | 0.62 | 0.44-0.89 |  | 1.04 | 0.73-1.48 |  | 0.47 | 0.30-0.75 |
| Racial Minority | 1.06 | 0.75-1.50 |  | 1.16 | 0.82-1.51 |  | 1.27 | 0.93-1.73 |  | 1.21 | 0.87-1.68 |
| Marital Status | 0.93 | 0.64-1.37 |  | 0.72 | 0.52-0.99 |  | 0.80 | 0.58-1.10 |  | 0.67 | 0.48-0.93 |
| Parental Status | 0.92 | 0.63-1.35 |  | 0.90 | 0.65-1.24 |  | 0.93 | 0.67-1.29 |  | 0.87 | 0.62-1.21 |
| Sexual Minority | 1.62 | 0.83-3.16 |  | 1.46 | 0.77-2.79 |  | 1.11 | 0.72-1.71 |  | 1.55 | 1.03-2.32 |
| Highest Edu | 0.71 | 0.47-1.07 |  | 0.64 | 0.44-0.93 |  | 0.76 | 0.55-1.06 |  | 0.61 | 0.43-0.85 |
| Navy v Army | 0.94 | 0.64-1.39 |  | 1.24 | 0.91-1.70 |  | 1.25 | 0.81-1.94 |  | 1.10 | 0.66-1.85 |
| Marine v Army | 1.04 | 0.73-1.48 |  | 0.80 | 0.57-1.13 |  | 1.07 | 0.79-1.44 |  | 1.28 | 0.94-1.74 |
| Air Force v Army | 0.83 | 0.53-1.28 |  | 0.72 | 0.48-1.07 |  | 0.77 | 0.55-1.07 |  | 0.74 | 0.51-1.07 |
| Rank | 0.49 | 0.21-1.13 |  | 0.46 | 0.21-1.03 |  | 0.50 | 0.24-1.06 |  | 0.32 | 0.13-0.80 |
| Warzone Deploy | 1.09 | 0.78-1.52 |  | 0.82 | 0.61-1.11 |  | 0.96 | 0.71-1.29 |  | 0.91 | 0.66-1.25 |
| MST | 1.51 | 0.91-2.50 |  | 1.27 | 0.77-2.08 |  | **1.64** | **1.16-2.33** |  | 1.18 | 0.82-1.71 |
| ACEs 1-2 v 0 | 0.83 | 0.60-1.13 |  | 0.98 | 0.74-1.29 |  | 0.97 | 0.72-1.32 |  | 0.96 | 0.70-1.33 |
| ACEs 3-4 v 0 | 1.05 | 0.76-1.44 |  | 1.19 | 0.90-1.59 |  | 0.99 | 0.73-1.34 |  | 0.78 | 0.57-1.07 |
| ACEs 5-6 v 0 | 1.31 | 0.93-1.85 |  | 0.90 | 0.63-1.28 |  | 0.94 | 0.66-1.35 |  | 0.99 | 0.70-1.39 |
| ACEs 7+ v 0 | 1.00 | 0.73-1.37 |  | 1.25 | 0.94-1.65 |  | 1.11 | 0.82-1.50 |  | 1.00 | 0.73-1.39 |
| PCL | **1.73** | **1.10-2.71** |  | 1.46 | 0.98-2.18 |  | **2.08** | **1.42-3.07** |  | **2.13** | **1.40-3.24** |
| PHQ | **2.23** | **1.31-3.78** |  | **3.55** | **2.25-5.60** |  | **2.05** | **1.28-3.26** |  | **1.79** | **1.12-2.88** |
| GAD | 1.40 | 0.87-2.23 |  | 1.32 | 0.86-2.03 |  | 1.07 | 0.69-1.64 |  | 1.07 | 0.69-1.65 |
| AUD | 1.03 | 0.74-1.44 |  | 1.15 | 0.86-1.55 |  | 1.22 | 0.90-1.65 |  | 1.23 | 0.88-1.71 |
| Attempt Pre Mil | **3.28** | **1.60-6.73** |  | **5.02** | **2.60-9.71** |  | **2.82** | **1.63-4.87** |  | **2.80** | **1.70-4.60** |
| Ideation Pre Mil | **2.46** | **1.51-4.01** |  | **1.71** | **1.07-2.72** |  | **2.37** | **1.44-3.91** |  | **3.48** | **2.23-5.42** |
| Witnessing | 0.71 | 0.47-1.06 |  | 0.76 | 0.53-1.11 |  | 1.40 | 0.90-2.19 |  | 0.91 | 0.59-1.40 |
| Perpetrating | **1.53** | **1.05-2.23** |  | **2.05** | **1.44-2.91** |  | 1.25 | 0.89-1.77 |  | 1.27 | 0.86-1.87 |
| Being Betrayed | **1.92** | **1.25-2.94** |  | 1.30 | 0.89-1.89 |  | **1.58** | **1.00-2.49** |  | **1.65** | **1.06-2.55** |
| *Note.* Participants were men (*n* = 8,809) and women (*n* = 5,248) veterans weighted to the population of post-9/11 veterans activated since 10/1/2001 and separated by 6/30/2015 (*N =* 3,691,536). Abbreviations include military sexual trauma (MST), adverse childhood events (ACEs), posttraumatic stress disorder (PCL), depressive disorder (PHQ), anxiety disorder (GAD), alcohol use disorder (AUD), and pre-military history of suicidal ideation (Ideation Pre Mil) and attempt (Attempt Pre Mil). All independent variables were binary (0, 1). Class variables were effect coded prior to analysis including age (ref: 20s), branch of service (ref: Army), and number of adverse childhood events (ref: 0). Bolding indicates at least small effect size (OR ≥ 1.52; Chen et al., 2010). | | | | | | | | | | | |

| Supplemental Table 2. *Gender-Stratified Weighted Rates of Suicide Attempt by Screening Status* | | | | |
| --- | --- | --- | --- | --- |
|  | Men | | Women | |
|  | Attempt During Service | Attempt After Separation | Attempt During Service | Attempt After Separation |
| MIES - Witnessing |  |  |  |  |
| Agree | 3.8 | 5.0 | 8.6 | 7.8 |
| Disagree | 1.7 | 2.1 | 2.5 | 3.6 |
| MIES – Perpetrating |  |  |  |  |
| Agree | 5.5 | 8.2 | 11.5 | 10.9 |
| Disagree | 1.7 | 2.1 | 3.9 | 4.3 |
| MIES – Being Betrayed |  |  |  |  |
| Agree | 4.9 | 6.1 | 9.3 | 8.9 |
| Disagree | 1.2 | 1.7 | 2.3 | 3.0 |
| Posttraumatic Stress Disorder |  |  |  |  |
| Positive | 7.7 | 10.1 | 14.5 | 14.6 |
| Negative | 1.4 | 1.8 | 2.6 | 2.9 |
| Depressive Disorder |  |  |  |  |
| Positive | 7.2 | 10.3 | 13.3 | 13.2 |
| Negative | 1.1 | 1.3 | 2.4 | 2.8 |
| Anxiety Disorder |  |  |  |  |
| Positive | 7.4 | 10.2 | 12.3 | 12.3 |
| Negative | 1.3 | 1.7 | 3.2 | 3.5 |
| Alcohol Use Disorder |  |  |  |  |
| Positive | 3.0 | 4.5 | 7.0 | 7.3 |
| Negative | 2.3 | 2.8 | 5.0 | 5.2 |
| Pre-Military Suicide Ideation |  |  |  |  |
| Yes | 9.9 | 11.9 | 18.1 | 21.9 |
| No | 2.1 | 2.8 | 3.8 | 3.4 |
| Pre-Military Suicide Attempt |  |  |  |  |
| Yes | 22.8 | 31.3 | 29.1 | 33.3 |
| No | 2.3 | 3.1 | 4.2 | 4.2 |
| Note. Values given as percentages | | | | |

| Supplemental Table 3. *Unadjusted, Partially Adjusted, and Fully Adjusted* *Weighted Estimates for the Association between Moral Injury and Suicide Attempt by Gender* | | | | | | | | | | | |
| --- | --- | --- | --- | --- | --- | --- | --- | --- | --- | --- | --- |
|  | Men | | | | |  | Women | | | | |
|  | Peri-Military Attempt | |  | Post-Military Attempt | |  | Peri-Military Attempt | |  | Post-Military Attempt | |
|  | AOR  (95% CI) | ARR  (95% CI) |  | AOR  (95% CI) | ARR  (95% CI) |  | AOR  (95% CI) | ARR  (95% CI) |  | AOR  (95% CI) | ARR  (95% CI) |
| *Unadjusted (Adjusted for Covariance between PMIE exposure by Witnessing, Perpetrating, and Being Betrayed; No Covariates)* | | | | | | | | | | | |
| Witness | 0.90  (0.62-1.32) | 0.90  (0.62-1.31) |  | 0.97  (0.67-1.40) | 0.97  (0.67-1.39) |  | **1.69**  **(1.14-2.50)** | **1.66**  **(1.14-2.41)** |  | 1.08  (0.73-1.59) | 1.08  (0.74-1.56) |
| Perpetration | **2.04**  **(1.43-2.92)** | **2.00**  **(1.42-2.82)** |  | **2.73**  **(1.95-3.83)** | **2.63**  **(1.91-3.62)** |  | **1.84**  **(1.33-2.55)** | **1.78**  **(1.31-2.40)** |  | **1.85**  **(1.31-2.62)** | **1.78**  **(1.29-2.45)** |
| Betrayal | **3.30**  **(2.17-5.02)** | **3.21**  **(2.14-4.79)** |  | **2.41**  **(1.69-3.45)** | **2.35**  **(1.67-3.31)** |  | **2.64**  **(1.76-3.97)** | **2.54**  **(1.73-3.72)** |  | **2.43**  **(1.65-3.57)** | **2.33**  **(1.62-3.31)** |
| *Partially Adjusted (Adjusted for Demographic and Military-Related Characteristics)* | | | | | | | | | | | |
| Witness | 0.82  (0.56-1.19) | 0.82  (0.56-1.19) |  | 0.88  (0.61-1.26) | 0.88  (0.62-1.25) |  | 1.45  (0.96-2.20) | 1.43  (0.96-1.19) |  | 0.96  (0.64-1.44) | 0.96  (0.65-1.42) |
| Perpetration | **1.83**  **(1.28-2.62)** | **1.80**  **(1.27-2.55)** |  | **2.45**  **(1.76-3.40)** | **2.37**  **(1.73-3.23)** |  | **1.58**  **(1.13-2.20)** | **1.55**  **(1.12-2.10)** |  | **1.54**  **(1.07-2.21)** | **1.50**  **(1.07-2.10)** |
| Betrayal | **2.64**  **(1.73-4.03)** | **2.59**  **(1.74-3.89)** |  | **1.89**  **(1.31-2.72)** | **1.86**  **(1.30-2.64)** |  | **1.90**  **(1.24-2.91)** | **1.86**  **(1.23-2.79)** |  | **1.90**  **(1.25-2.89)** | **1.85**  **(1.24-2.73)** |
| *Fully Adjusted (Adjusted for Demographic/Military-related Characteristics, Current Mental Health, and History of Suicidal Thoughts and Behaviors)* | | | | | | | | | | | |
| Witness | 0.71  (0.48-1.06) | 0.71  (0.48-1.06) |  | 0.76  (0.53-1.11) | 0.76  (0.54-1.11) |  | 1.40  (0.90-2.19) | 1.39  (0.90-2.13) |  | 0.91  (0.59-1.40) | 0.91  (0.60-1.38) |
| Perpetration | **1.53**  **(1.05-2.23)** | **1.52**  **(1.05-2.18)** |  | **2.05**  **(1.44-2.91)** | **2.01**  **(1.43-2.80)** |  | 1.25  (0.89-1.77) | 1.24  (0.89-1.72) |  | 1.27  (0.86-1.87) | 1.26  (0.87-1.80) |
| Betrayal | **1.92**  **(1.25-2.94)** | **1.90**  **(1.25-2.87)** |  | 1.30  (0.90-1.89) | 1.29  (0.89-1.86) |  | **1.58**  **(1.00-2.49)** | **1.56**  **(1.00-2.41)** |  | **1.65**  **(1.06-2.55)** | **1.62**  **(1.06-2.44)** |
| *Note.* Participants were men (*n* = 8,809) and women (*n* = 5,248) veterans weighted to the population of post-9/11 veterans activated since 10/1/2001 and separated by 6/30/2015 (*N =* 3,691,536). Partially adjusted models account for sociodemographic and military characteristics. Fully adjusted models account for sociodemographic and military characteristics, mental health outcomes, and history of suicidal self-directed violence. Exposure to potentially morally injurious events was modeled by witnessing (0 = disagree, 1 = agree), perpetrating (0 = disagree, 1 = agree), and being betrayed (0 = disagree, 1 = agree). Bolding indicates at least small effect size (OR ≥ 1.52; Chen et al., 2010). | | | | | | | | | | | |
